# Factors Associated with the Consumption of Indigenous Crops Among Farming Households in KwaZulu-Natal, South Africa

**DOI:** 10.3390/foods14071092

**Published:** 2025-03-21

**Authors:** Nomfundo Shelembe, Simphiwe Innocentia Hlatshwayo, Albert Thembinkosi Modi, Tafadzwanashe Mabhaudhi, Mjabuliseni Simon Cloapas Ngidi

**Affiliations:** 1 Centre for Transformative Agricultural and Food Systems, School of Agricultural, Earth and Environmental Sciences, College of Agriculture, Engineering and Science, University of KwaZulu-Natal, Private Bag X01, Scottsville, Pietermaritzburg 3201, South Africa; hlatshwayos2@ukzn.ac.za (S.I.H.); mabhaudhi@ukzn.ac.za (T.M.); 2 African Centre for Food Security, School of Agricultural, Earth and Environmental Sciences, College of Agriculture, Engineering and Science, University of KwaZulu-Natal, Private Bag X01, Scottsville, Pietermaritzburg 3201, South Africa; 3 Faculty of Natural Sciences, Walter Sisulu University, Private Bag X1, Mthatha 5099, South Africa; amodi@wsu.ac.za; 4 Centre on Climate Change and Planetary Health, London School of Hygiene & Tropical Medicine, London WC1E 7HT, UK; 5 Department of Agricultural Extension and Rural Resource Management, School of Agricultural, Earth and Environmental Sciences, College of Agriculture, Engineering and Science, University of KwaZulu-Natal, Private Bag X01, Scottsville, Pietermaritzburg 3201, South Africa

**Keywords:** exotic crops, indigenous crops, consumption, binomial regression

## Abstract

South African farming households face several challenges regarding food security, poverty, micronutrient deficiencies and hidden hunger. This is due to millions of households lacking access to food and an adequate food basket. Consumption of indigenous crops has been proposed to help sustain vulnerable households since these crops have low production costs and are climate-resilient. However, research has found the consumption of these crops across South Africa to be relatively low. This study aims to examine the factors associated with the consumption of indigenous crops among farming households in the KwaZulu-Natal province of South Africa. A sample of 260 farming households was selected using simple random sampling. The results showed that farmers commonly cultivate exotic crops, such as spinach, cabbage, carrot, and butternut, more than any indigenous crops, except for a few cultivating taro and sweet potato. The binomial logit regression results revealed that an increase in the number of females and children within a household and farmers’ experience increased the likelihood of consuming indigenous crops, whilst monthly food expenses decreased the likelihood of consuming indigenous crops. There is a considerable gap between the consumption and production of indigenous crops. The findings established that although many farming households indicated that they consume indigenous crops, this was not reflected in their cultivated crops. The study also concluded that farming households may be more aware of the nutritional benefits of indigenous crops, since an increase in the number of children in a household was linked to an increase in consumption of these crops. Additionally, experience in farming is vital, as it increases the consumption of indigenous crops. The study recommends government interventions that include increasing the production of indigenous crops by including them alongside the cultivation of exotic crops. Future work should also focus on awareness programs to promote the nutritional benefits of consuming indigenous crops. This, coupled with training centered on indigenous crops, could incentivize farming households to cultivate more of these crops for easier access.

## 1. Introduction

Global food production is at a critical point, with meeting the Sustainable Development Goals (SDGs) by 2030, especially SDG 2, seeming increasingly unlikely [1]. This goal pressures scientists and practitioners to find sustainable ways to ensure the supply of nutritious food without harming the environment. One approach that is gaining traction internationally is the increased use of indigenous crops, sustainable food systems, and supply chains while boosting consumer demand for these crops [2]. Over the years, research and agricultural subsidies have favored a few dominant crops—wheat, rice, and maize [3]—which now account for 51% of global caloric consumption [4]. As market incentives encourage the shift to exotic crops, many farmers have abandoned indigenous varieties [5]. However, relying solely on these crops cannot provide a balanced diet, as demonstrated by countries like Brazil, Kenya, and India, where indigenous crops are being reintegrated into food security policies to diversify farming systems and promote their adoption [6].

In South Africa, despite several factors working in its favor on the agricultural front, millions of individuals lack access to nutritious and safe food [7]. This is primarily due to poverty, which is especially prevalent in rural areas. In the KwaZulu-Natal province, although diverse food options are available in rural areas, indigenous crops have often been overlooked, further reducing the food security base. This has resulted in initiatives aimed at addressing food insecurity that continue to overlook indigenous crops, thus diminishing the diversity of the food supply. Despite being underappreciated, considerable evidence from the literature highlights that indigenous crops play a vital role in combating hunger and food insecurity [8,9]. In KwaZulu-Natal, as in other regions, these crops could provide a sustainable solution to improving food security and nutritional intake, given their resilience and adaptability to local conditions.

Indigenous crops contribute significantly to local economies and diets by promoting biodiversity, preserving local knowledge, and providing household income [10,11]. While their benefits are recognized in other African countries, their use in South Africa has declined due to poverty [12,13], limited resources, and low self-esteem among rural black communities [2,14]. Additionally, factors like legislation and technology have contributed to this decline. Studies have shown that shifting from indigenous crops to exotic crops increases health risks, including malnutrition and non-communicable diseases, particularly in vulnerable households [7,15,16]. Affording a healthy food basket is a major concern for South African households, with low-income families spending around 35% of their income on food while remaining vulnerable to malnutrition [17,18]. Increased reliance on food purchases discourages home production and exacerbates food insecurity [19,20]. Research suggests that promoting the consumption of indigenous crops, which are more affordable and climate-resilient, could help alleviate these challenges [21].

Despite the recognized nutritional and medicinal benefits of indigenous crops, their consumption remains low compared to exotic crops like spinach and cabbage [22,23]. The displacement of indigenous crops by major exotic varieties has contributed to challenges in global food systems, particularly in underdeveloped regions [24]. Incorporating indigenous crops into South Africa’s food systems could help diversify rural economies and improve food security [25]. However, existing studies on indigenous crop consumption in South Africa are outdated, with research over 10 years old focused on provinces like Limpopo and Northwest [12,26]. KwaZulu-Natal, despite having many agricultural households, faces ongoing food insecurity, and there is a lack of literature focusing on the production, consumption, and market value chain for indigenous crops in the province. This study fills this gap by examining the factors associated with the consumption of indigenous crops in the KwaZulu-Natal province of South Africa, providing current, valuable insights for policymakers, producers, and consumers to enhance the cultivation, consumption, and market integration of these crops, ultimately helping reduce food insecurity in rural and peri-urban areas.

## 2. Materials and Methods

**Figure 1:**
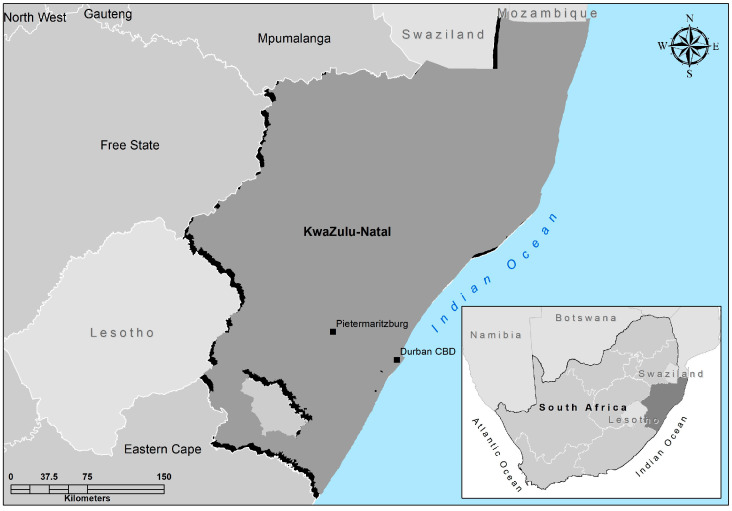
A map showing the KwaZulu-Natal study area.

The study was conducted in the KZN province of South Africa (Figure 1), which is characterized by a subtropical coastline, grasslands in the east, and a wide mountain range in the west. It has a varied climate and diverse topography [27]. The KwaZulu-Natal province has the second-largest projected population in South Africa, where agriculture is considered a noticeable contributor to household food security among vulnerable households [28].

The KwaZulu-Natal province consists of 11 districts, from which uMgungundlovu, eThekwini, and Harry Gwala were selected based on three key criteria: (1) their representation of diverse agro-ecological zones to capture variations in indigenous crop cultivation; (2) their inclusion of both rural and peri-urban farming communities; and (3) their distinctive agricultural profiles—uMgungundlovu offering mixed urban-rural farming systems, eThekwini providing peri-urban farming contexts, and Harry Gwala representing traditional rural farming with strong indigenous knowledge systems.

The districts selected had a high number of rural and peri-urban households that participate in farming; however, most households are dependent on the South African social grant, making them economically vulnerable due to their limited nature. Additionally, the districts selected have been found to be significantly food insecure [7]. Studies that add to the benefits of the inclusion of indigenous crops in household farming systems can be helpful in the improvement of food security among vulnerable households. Furthermore, the study areas were chosen because there was a gap in the literature of studies focusing on indigenous crop consumption in KwaZulu-Natal. After district selection, five areas within the districts were randomly selected and sampling frames were sourced. These areas included Mbumbulu, Swayimane, Imbali, Shayamoya, and Cabazi locations.

This study employed a quantitative research design utilizing structured questionnaires as the primary data collection instrument. This methodological approach aligns with previous agricultural research studies that have successfully implemented survey-based methods to examine various aspects of farming households [2,7,8]. Data were collected through face-to-face interviews administered by trained enumerators in IsiZulu, the predominant language in the study areas. The questionnaire encompassed multiple domains, including socioeconomic characteristics, perceptions of indigenous crops, cultivation patterns, consumption behaviors, and household-level interactions with indigenous crops.

Simple random sampling was employed to select participants from a sampling frame of a total of 567 farming households, ensuring equal probability of selection for each household. The sample size was determined using a 95% confidence interval and a 5% margin of error. This sampling approach, coupled with the specified confidence interval and margin of error, was chosen to optimize statistical reliability while ensuring representative coverage of the study area, though acknowledging the inherent limitations of sampling a subset of the total population. Using a higher confidence interval, such as 95%, requires a larger sample size. However, if a lower confidence interval, such as 80%, is used, the sample size will significantly decrease. This is similar to the margin of error, where 5% is commonly used because a lower margin of error requires a larger sample size to be more accurate.

The sample size was calculated using the Cochran formula with a Z-value of 1.96 (95% confidence level) and a margin of error of 0.05. After applying the finite population correction for our known population of 567 households, the minimum required sample was 229. We increased this to 260 to account for potential non-responses and ensure adequate district representation. The use of a 95% confidence interval and a 5% margin of error resulted in 260 farming households distributed across three districts: uMgungundlovu (n = 120), Harry Gwala (n = 80), and eThekwini (n = 60).

## 3. Analytical Framework

### 3.1. Binomial Logit Regression to Investigate the Determinants of Consumption of Indigenous Crops

A binomial logit regression was selected to investigate the predictor variables that determine the consumption of indigenous crops. The dependent variable, consumption of indigenous crops, had a binary nature, taking a value of 0 for farming households who consume indigenous crops and 1 if they do not. The regression model was chosen as suitable for the statistical technique, and the factors that determine whether farming households consume or do not consume indigenous crops were analyzed. According to Maharjan and Joshi [29], a binomial logit regression model is considered the appropriate model for socioeconomic analysis in which both continuous and categorical independent variables are applicable. Harris et al. [30] stated two primary reasons for the researcher’s use of logistic regression. Initially, the logistic model imposes a threshold and interaction effects, and allows for examining social interaction [30,31]. The binomial logit regression has been used in several food security-based research to investigate determinants of household food security and technology adoption by farmers [32,33,34]. The binomial logistic regression was found to be ideal for studying the factors associated with indigenous crop consumption among farming households in KwaZulu-Natal as it effectively analyzes dichotomous outcomes. The model expresses results as odds ratios, allowing for a clear interpretation of how various factors influence consumption likelihood. The model’s ability to accommodate various independent variable types, such as continuous and categorical, allows for the examination of relevant socioeconomic, agricultural, cultural, market-related, and policy factors specific to the KwaZulu-Natal context. This approach will then enable the identification of targeted interventions to promote these crops.

The probability of a household consuming indigenous crops depends on vectors of independent variables *Xi* and a vector of unknown parameter β; the vector X*i* represents farming households’ socioeconomic and other factors.

The mathematical formulation of the binomial logistic model is as follows:

(1) $$Pi=E\left(Y=1/Xi=\frac{1}{1+e^{\left(\beta 0+\beta_{1}X1\right)}}\right)$$

The equation of the probability of a farming household consuming indigenous crops can be expressed by

(2) $$Pi=\frac{1}{1+e^{-Z}}$$

While the probability to not consume indigenous crops can be expressed by

(3) $$1-Pi=\frac{1}{1+e^{Z}}$$

Overall, this can be expressed as

(4) $$\frac{Pi}{1-Pi}=\frac{1+e^{Z}}{1+e^{-Z}}$$

Referencing from above, (*Pi*/1 − *Pi*) is the odds ratio representing the farming households that consume indigenous crops. Using the natural log of the above equation, it is then determined that

(5) $$Li=ln\frac{Pi}{1-Pi}=Zi=\beta o+\beta lXl+\beta 2\times 2+\cdots +\beta nXn$$

where *Pi* = is the probability that a farming household consumes indigenous crops, and *Ζi* = is the function of the explanatory variables (*Xi*), which is also expressed as *β*_0_, the intercept.

*β*_1_, *β*_2_………*βn* are slopes of the equation in the model.

*Li* = is the log of odds ratio, which is not only linear in *Xi* but also linear in the parameters.

The independent variables used in the model are represented in Table 1 below.

### 3.2. Justification for Proposed Variables

The number of children refers to the number of children in each household and was captured as a continuous variable. Children need specific food and a nutritional diet to survive. This increases food costs within a household and exposes low-income households to various shocks and food insecurity. Hence, indigenous crops are a way to mitigate household food insecurity among children as they are highly nutritious and can be cooked in several ways. Mbhenyane [35] noted that children who consistently consume these crops were less vulnerable to hidden hunger. The number of children was hypothesized to positively influence the consumption of indigenous crops.

The number of females refers to the number of females within a given households and was captured as a continuous variable. Several studies have found females to produce and consume indigenous crops in different countries, particularly in Sub-Saharan Africa [36,37]. The number of females was hypothesized to positively influence the consumption of indigenous crops.

The number of males refers to the number of males within a given households and was captured as a continuous variable. Studies have found different results considering males’ consumption of indigenous crops in South Africa and Africa. Zulu et al. [38] found that males’ consumption of indigenous crops was correlated with their location, where males residing in rural areas consumed them, and those in urban areas did not. Contrastingly, Akpa et al. [39] found that males consume indigenous crops as much as females. The number of males was hypothesized to have either a positive or negative influence on the consumption of indigenous crops.

Total household income refers to the total amount of income incurred by a given household monthly and was captured as a categorical variable. Income is one of the main drivers in a household food basket, and indigenous crops can be used to supplement food baskets. However, high-income farming households generally do not consume indigenous crops as they may prefer exotic crops [40,41]. Hence, the total household income variable was hypothesized to have a negative influence on the consumption of indigenous crops.

Educational level refers to the level of education a household head has achieved and was captured as a categorical variable. Farming households who are more educated will likely consume indigenous crops for their nutritional benefits [26,42]. The education level variable was hypothesized to positively influence the consumption of indigenous crops.

Monthly food expenses refer to a household’s monthly food costs and were captured as a categorical variable. The consumption of indigenous crops differs among low- and high-income farming households. Households with a higher number of household members will require a higher amount of food, and this is a challenge for low-income farming households [26]. Indigenous crops may also be used as an alternative in times of food shortages [16]. An increase in monthly food expenses was hypothesized to negatively influence the consumption of indigenous crops.

Farming Period refers to the years a farmer has participated in agriculture and was captured as a categorical variable. An increase in farming experience increases the likelihood of consumption as these farmers are more aware of the costs and benefits of these crops [43]. The farming experience variable was hypothesized to positively influence the consumption of indigenous crops.

Perceived marketing potential refers to the perceived value of indigenous crops in the market and was captured as a categorical variable. Whether farming households find indigenous crops of great value depends on their perception and knowledge of these crops. Studies have shown that indigenous crops carry a negative perception of being poor man’s food [2,15,41,44]. The perceived marketing potential variable was hypothesized to have either a positive or negative influence on the consumption of indigenous crops.

## 4. Results and Discussion

### 4.1. Cultivated Versus Purchased Crops Among Farming Households

Participants were asked which crops they commonly cultivated throughout the year. Within the sample size, 63.8% of farming households cultivated exotic crops such as maize, cabbage, beans, spinach, and potatoes. Other common vegetables produced by the farmers were onions, carrots, green peppers, chilies, butternut, sugarcane, and beetroot; 23.5% grew a mix of exotic and indigenous crops, which included sweet potato, intufeshe (wild kale/Ethiopian kale) and amadumbe (taro). Some farming households grew taro or sweet potatoes, with a small number cultivating imbuya (amaranthus) and intufeshe (wild kale/Ethiopian kale).

Exotic crops (primarily maize, cabbage, and spinach) constituted the predominant cultivation category, followed by mixed cultivation systems incorporating exotic and indigenous crops (including sweet potato and taro). These findings align with previous research indicating that introducing exotic crop varieties in Africa has led to decreased production and consumption of indigenous crops [45,46]. Analysis of cultivation purposes revealed that 62% of farmers grew crops primarily for household consumption, while only 5% cultivated exclusively for market sales. Mixed-purpose cultivation (food and market) accounted for 19.5% of respondents, with 13.6% reporting cultivation as a recreational activity. This distribution pattern suggests a disconnect between indigenous crop consumption preferences and actual cultivation practices, with farmers demonstrating a stronger inclination toward exotic crop production. The data further indicate that farmers prioritize exotic crops for consumption and commercial purposes. The preference to cultivate exotic crops rather than indigenous crops suggested that farming households have more prior knowledge of exotic crops in terms of their potential financial benefits and market demand for exotic crops. This is because farming households commonly experience pressure to maintain consistent profit and exotic crops have established markets all over country. This preference aligns with Mabhaudhi et al. [2], who identified market appeal and consumer preferences as key drivers for farmers’ emphasis on exotic over indigenous crop cultivation in South Africa. The results also coincide with those of Onomu [5] where indigenous foods were reported to have less market participation in African markets due to insufficient knowledge of their potential.

An analysis of purchasing patterns revealed that 92.6% of farming households continued to source vegetables from supermarkets despite maintaining their agricultural production. This high percentage of market dependence among active farming households is particularly noteworthy. The most commonly purchased items included butternut, tomatoes, spinach, and cabbage. This paradoxical relationship between cultivation and purchasing behavior suggests several potential underlying factors affecting agricultural productivity, which may include the following:

- Insufficient yield from current cultivation practices to meet household demands;
- Limited crop diversification due to monocropping practices, creating gaps in household food availability;
- Inadequate access to agricultural knowledge and support systems necessary for successful farming and market participation.

These findings corroborate the observations of Coetzer and Breedeveld [19], who documented the persistent reliance of South African farming households on supermarkets despite their engagement in agricultural activities. The data indicate a complex relationship between household farming practices and formal market dependence, suggesting opportunities for interventions to enhance agricultural self-sufficiency. Similarly, Koppmair et al. [17] reported that farming households from Malawi continue to purchase vegetables from supermarkets due to insufficient storage facilities and inputs to cultivate throughout the year.

### 4.2. Consumption of Indigenous Crops

**Figure 2:**
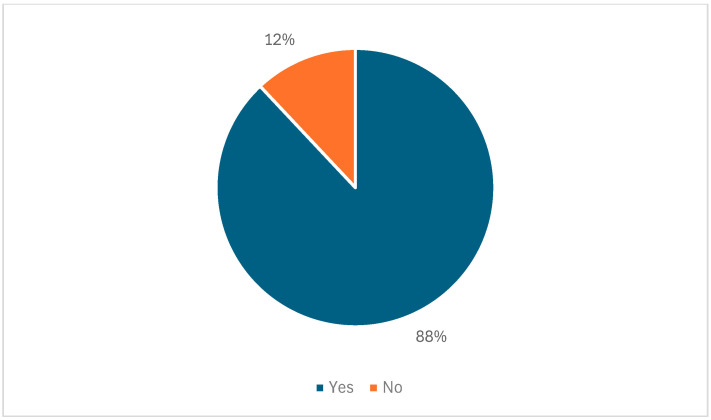
Consumption rate of indigenous crops among farming households in KwaZulu-Natal.

Participants were asked about their consumption preferences regarding indigenous crops, as illustrated in Figure 2. The results indicated that 88% of farming households reported consuming indigenous crops, while 12% did not. This higher proportion of farming households consuming indigenous crops suggests that a greater number of households may now be more aware of the potential benefits these crops offer. However, these findings were somewhat surprising and in contrast to previous research on the consumption of indigenous crops in South Africa, which has typically shown lower consumption rates.

The current results challenge past studies, which indicated that the consumption of indigenous crops in South Africa was relatively low [12,26,34,35]. Prior research attributed this low consumption to several factors, including a limited perception of the value of indigenous crops and a lack of awareness about their potential health benefits. In contrast, the higher consumption observed in the current study may reflect a shift in attitudes or an increasing recognition of the role indigenous crops can play in enhancing dietary diversity.

This shift could suggest that farming households are becoming more proactive in diversifying their diets, potentially driven by growing awareness of the nutritional and ecological advantages of indigenous crops. Such a change in consumption patterns may also reflect broader trends toward sustainability and food security, which are increasingly recognized as critical elements in agricultural practices.

**Figure 3:**
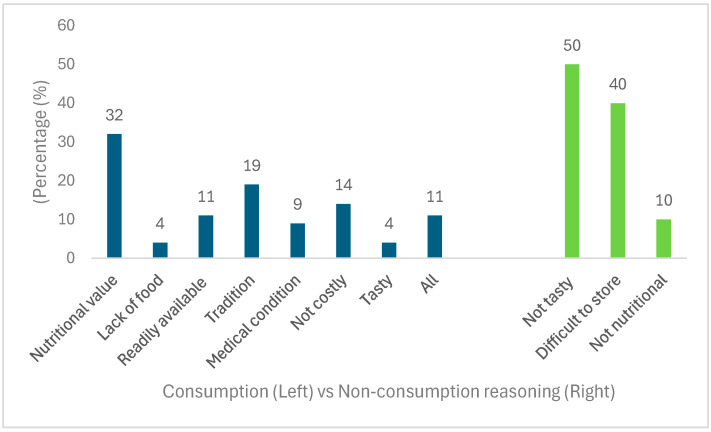
Reasons for indigenous crop consumption and non-consumption rate among farming households in KwaZulu-Natal.

Figure 3 shown below, further shows the reasoning behind the consumption and non-consumption of these crops. The highest reason for consumption was the perception of their nutritional value (32%), followed by tradition (19%). Participants also reported these crops as not costly (14%), readily available (11%), assisting with medical conditions (9%), assisting in lack of food (4%), tasty (4%), and all of the above (11%). The results further show a more positive relationship building with indigenous crops among rural and peri-urban areas, as the reasoning for consumption in prior years was mainly due to lack of food [47,48,49]. However, it must be noted that the results indicated that farming households need to be made aware of these crops’ adaptive components (i.e., water efficiency and adaptability to different environmental conditions).

Participants provided several reasons for not consuming indigenous crops, including taste (50%), low nutritional value (10%), and difficulty in storing them for long periods (40%). These reasons were further explained by the way farming households perceive these crops. When asked who they believed should consume indigenous crops, the results indicated that older women (38.8%) were most commonly viewed as the most suitable group to consume them, followed by all individuals (35.4%), older men (18.8%), young men (5%), and a small number selecting young women (1.9%).

The results suggest a somewhat positive perception of indigenous crops, as all individuals were listed as the second-highest choice for consumption. However, the slightly higher preference for older women suggests that they are seen as the primary group for consuming these crops. This finding is not surprising and likely reflects the role of older women as custodians of indigenous knowledge, with the use of indigenous crops being an integral part of this knowledge. In rural and peri-urban communities, older women often serve as key figures in educating younger generations about the importance of indigenous crops [50].

Additionally, the results may reflect the common observation that older women are more likely to consume these crops due to their perceived health benefits. This aligns with past findings from other regions of South Africa, such as the Eastern Cape province, where studies have shown that women, in comparison to men, are more likely to utilize indigenous crops for both food and medicinal purposes [15,26,50].

### 4.3. Determinants of Indigenous Crops’ Consumption Among Farming Households

The binomial logit regression model was employed to assess the determinants of indigenous crop consumption among farming households. The dependent variable was binary, where participants indicated whether or not they consumed indigenous crops. Table 2 presents the results of the binomial logit regression, with the reference category set as “consumes indigenous crops”. Before estimating the selection model, the potential for multicollinearity was examined using the variance inflation factor (VIF). Testing for multicollinearity is crucial as it helps improve the reliability of inferences drawn from the model. In this study, multicollinearity was assessed through the VIF, with a value greater than ten indicating a multicollinearity problem among the independent variables. All variables in the analysis were found to have VIF values below 10, suggesting no significant multicollinearity issues. The overall model was statistically significant (*p* = 0.000), and the Nagelkerke R-squared value was 0.373, indicating that the independent variables explained 37.3% of the variability in the dependent variable. The Hosmer and Lemeshow test was non-significant (*p* = 0.4264), as expected in binary logistic regression, indicating a good model fit. To understand how the independent variables influence the consumption of indigenous crops, both the coefficients and odds ratios were analyzed. Only the significant variables are discussed in Table 2 below.

The binomial logit regression results reveal key factors influencing the consumption of indigenous crops among farming households. A significant and positive relationship was found between the number of females in the household and the likelihood of consuming indigenous crops (coefficient = 0.497, *p* = 0.018)

The number of females variable had a significant (0.018) and positive coefficient (0.497). All other factors held constant; an increase in the number of females within a household by one unit increased the likelihood of consuming indigenous crops by 0.497 units. This suggests the central role that women play in promoting and preserving traditional food practices due to various factors. These may include (1) women’s role in food preparation and management, as in the Zulu culture and many other cultures, women are primarily responsible for cooking and managing household food. As such, they might be more inclined to use indigenous crops, which are often nutritious. (2) Women’s knowledge and transmission of traditional practices may be another factor; women might be more likely to possess and pass down knowledge about indigenous crops, their preparation, and their cultural significance to future generations. This aligns with results from Ayinde et al. [51] and Atuna et al. [52], who reported that females consume indigenous crops more than males. The results also coincided with those of Bhengu [36], who found that females consume considerably more indigenous crops. Furthermore, Maltitz and Bahta [53] reported that an increase in the consumption of indigenous crops by women fosters empowerment and overall household food security. This finding could suggest that targeting women in agricultural extension programs and nutrition education could promote the use of indigenous crops. Likewise, supporting women’s groups and cooperatives could preserve and promote traditional food practices. There may be a need to encourage male involvement in food preparation and management to promote greater sharing of responsibilities and knowledge.

As indicated by the regression results, households with more children (coefficient = 0.610, *p* = 0.012) also show a higher likelihood of consuming indigenous crops. This suggest that larger rural households with more children might prioritize food security and rely on indigenous crops. This finding highlights the critical role that food security plays in larger households. The presence of children often drives caregivers to prioritize the nutritional quality of meals, and indigenous crops, which are both resilient and nutritious, become an important part of the household diet. This could also mean that children are likely to be taught about and socialized to consume indigenous crops by their parents, perpetuating the consumption of indigenous crops or food. Based on these findings, interventions on nutrition education and agricultural extension programs could prioritize or target households with children to promote the consumption of indigenous crops. Programs could engage children in activities like gardening, cooking, or food preservation to promote their interest in and knowledge of indigenous crops.

These results coincide with several studies where an increase in the consumption of indigenous crops among children was associated with good nutritional status [13,54,55].

The association between monthly food expenses and a decreased likelihood of consuming indigenous crops (coefficient = −4.015, *p* = 0.041) is particularly noteworthy. An increase in the monthly income spent on a household food basket decreased the likelihood of a household consuming indigenous crops by 4.015 units. This suggests that as income rises, households might adopt more Westernized or urbanized food preferences, leading to a decline in the consumption of indigenous crops. Higher-income households might have better access to modern markets and supermarkets, making it easier for them to purchase non-indigenous crops. Notwithstanding the challenges of COVID-19 leaving a number of South Africans unemployed, these results are expected because over sixty percent of South Africans generally consumed and purchased foods from well-organized supermarkets around the country [40,56]. Higher-income households often have greater access to supermarkets and processed foods, making indigenous crops less appealing. While this is a concerning trend, it also presents an opportunity for future interventions. Policymakers should consider strategies to make indigenous crops more accessible and attractive to higher-income households. Future interventions could focus on making indigenous crops more accessible and appealing to higher-income households by introducing them into modern markets and retail or by highlighting their environmental and nutritional benefits. Furthermore, future interventions such as programs promoting indigenous crops could prioritize lower-income households, which might be more likely to adopt these crops due to economic constraints.

The study also found that farming experience significantly increased the likelihood of indigenous crop consumption (coefficient = 4.643, *p* = 0.006). The findings imply that experienced farmers are likely more familiar with the cultivation and benefits of indigenous crops and may choose to incorporate them into their diets due to their lower input requirements and nutritional value. These results concur with those of Andani et al. [43] and Melash et al. [57], who noted that farmers with a higher level of experience had a greater likelihood of utilizing indigenous crops. Farmers are also likely to have better access to indigenous crops, either through their own farming activities or through local markets and networks. In addition, farmers with experience might have a stronger cultural connection to indigenous crops, which could influence their food choices. Based on these findings, future interventions could focus on leveraging the knowledge and experience of farmers to promote indigenous crops.

## 5. Conclusions and Recommendations

The study’s objective was to analyze the different factors associated with the consumption of indigenous crops among farming households. The results showed that farmers commonly cultivated exotic crops such as spinach, cabbage, carrot, and butternut more than any indigenous crops, except for a few cultivating taro and sweet potato. The binomial logit regression results revealed that the number of females and children within a household and the farmer’s experience increased the likelihood of consuming indigenous crops, whilst monthly food expenses decreased the likelihood of consuming indigenous crops.

There is a huge gap between the consumption and production of indigenous crops. The study concluded that although many farming households indicated that they consume indigenous crops, this was not reflected in their cultivated crops. It was also concluded that when households have children, there is a great need to secure nutritional food, and caregivers turn to indigenous crops. Furthermore, experience in farming is vital, as it increases the consumption of indigenous crops. Therefore, the study recognizes the need for increased awareness and recommends that farming households adopt many climate-adapting indigenous crops for cultivation and consumption.

Based on these findings, we propose the following specific recommendations for policymakers and stakeholders:

1. Educational and awareness initiatives
  - Develop structured training programs for farmers, focusing on indigenous crop cultivation techniques;
  - Implement school-based educational programs to familiarize younger generations with indigenous crops;
  - Create multimedia awareness campaigns highlighting the nutritional and economic benefits of indigenous crops;
  - Establish demonstration farms in communities to showcase successful indigenous crop cultivation.
2. Market development and economic support
  - Create dedicated market spaces for indigenous crop trading;
  - Develop value-addition programs for indigenous crop products;
  - Establish price support mechanisms for indigenous crop producers;
  - Facilitate connections between farmers and institutional buyers (schools, hospitals, and prisons).

Study limitations. This research was conducted exclusively in KwaZulu-Natal, South Africa, and its findings may not be generalizable to other regions or countries. Further research across different geographical and cultural contexts is recommended to develop a more comprehensive understanding and policies.

## Figures and Tables

**Table 1:** Variables included in the model.

Independent Variable	Type of Measurement	Categorical Explanation	Prior Expectation
Number of children in household	Continuous		+
Number of males in a household	Continuous		−/+
Number of females in a household	Continuous		+
Total monthly household income	Categorical	0 = R0 to R500 1 = R501 to R1000 2 = R1001 to R1500 3 = R1501 to R2500 4 = R2501 to R3500 5 = R3501 to R4500 6 = Greater than R4500	−
Education Level	Categorical	0 = None 1 = Primary 2 = Secondary 3 = Tertiary	+
Monthly household food costs	Categorical	0 = R0 to R500 1 = R501 to R1000 2 = R1001 to R1500 3 = R1501 to R2500 4 = Greater than R2500	−
Farming Period	Categorial	0 = Under 4 years 1 = 4–10 years 2 = 10–20 years 3 = Greater than 20 years	+
Indigenous crop marketing potential	Categorical	0 = Yes 1 = No	−/+

**Table 2:** Determinants of indigenous crops’ consumption among farming households: ^1^ beta, the coefficient and the amount in units by which the dependent variable increases when increasing the independent variable; ^2^ standard error; ^3^ *p* ≤ 0.05; ^4^ exponential beta, the predicted change in odds for a unit increase of the dependent variable.

Variables	Coefficient ^1^	Standard Error ^2^	*p* > z ^3^	Exponential Beta ^4^
Number of males in the household	−0.177	0.205	0.389	0.838
Number of females in a household	0.497	0.210	0.018 **	0.608
Number of children in a household	0.610	0.243	0.012 **	1.840
Total Household Income				
R0 to R500	Reference		0.053 *	
R501 to R1000	0.329	1.031	0.750	1.390
R1001 to R1500	1.280	1.159	0.269	3.597
R1501 to R2500	−0.985	1.118	0.378	0.373
R2501 to R3500	−2.600	1.633	0.111	0.074
R3501 to R4500	0.785	1.352	0.561	2.193
Greater than R4500	1.073	1.347	0.426	2.923
Education Level				
None	Reference		0.227	
Primary	−0.465	1.271	0.714	0.628
Secondary	−1.502	1.207	0.213	0.223
Tertiary	−2.171	1.356	0.110	0.114
Monthly Food Expenses				
R0 to R500	Reference		0.015 **	
R501 to R1000	1.011	0.788	0.200	2.749
R1001 to R1500	1.677	1.127	0.137	5.348
R1501 to R2500	−0.499	1.105	0.651	0.607
Greater than R2500	−4.015	1.963	0.041 **	0.018
Farming Period				
Under four years	Reference		0.041 **	
4 to 10 years	0.699	0.751	0.352	2.011
10 to 20 years	1.227	0.855	0.151	3.412
Greater than 20 years	4.643	1.691	0.006 ***	103.834
Indigenous crops: Marketable	Reference			
Indigenous crops: Unmarketable	−0.006	0.543	0.990	0.994
Constant	2.629	1.520	0.084 *	13.856

Mean VIF	2.80			
Prob > Chi^2^	0.000			
Hosmer and Lemeshow	0.4264			
Nagelberke R^2^	0.373			
Number of observations	260			

* = significant at the 0.1 level; ** = significant at the 0.05 level; *** = significant at the 0.01 level.

## Data Availability

The original contributions presented in this study are included in the article. Further inquiries can be directed to the corresponding authors.
